# The TOX–RAGE axis mediates inflammatory activation and lung injury in severe pulmonary infectious diseases

**DOI:** 10.1073/pnas.2319322121

**Published:** 2024-06-20

**Authors:** Hyelim Kim, Hee Ho Park, Hong Nam Kim, Donghyuk Seo, Kyung Soo Hong, Jong Geol Jang, Eun U Seo, In-Young Kim, So-Young Jeon, Boram Son, Seong-Woo Cho, Wantae Kim, June Hong Ahn, Wonhwa Lee

**Affiliations:** ^a^Brain Science Institute, Korea Institute of Science and Technology, Seoul 02792, Republic of Korea; ^b^Department of Biotechnology, Yonsei University, Seoul 03722, Republic of Korea; ^c^Department of Bioengineering, Hanyang University, Seoul 04763, Republic of Korea; ^d^Research Institute for Convergence of Basic Science, Hanyang University, Seoul 04763, Republic of Korea; ^e^Division of Bio-Medical Science and Technology (Korea Institute of Science and Technology School), Korea University of Science and Technology, Seoul 02792, Republic of Korea; ^f^School of Mechanical Engineering, Yonsei University, Seoul 03722, Republic of Korea; ^g^Yonsei-Korea Institute of Science and Technology Convergence Research Institute, Yonsei University, Seoul 03722, Republic of Korea; ^h^Department of Chemistry, Sungkyunkwan University, Suwon 16419, Republic of Korea; ^i^Division of Pulmonology and Allergy, Department of Internal Medicine, College of Medicine, Yeungnam University and Regional Center for Respiratory Diseases, Yeungnam University Medical Center, Daegu 42415, Republic of Korea; ^j^Department of Life Science, University of Seoul, Seoul 02504, Republic of Korea

**Keywords:** TOX, RAGE, severe COVID-19, septic shock, fibroproliferative ARDS

## Abstract

Thymocyte selection–associated high-mobility group box (TOX) is a transcription factor that is crucial for T cell exhaustion during chronic antigenic stimulation, but its role in inflammation is poorly understood. Here, we report that TOX extracellularly mediates drastic inflammation upon severe acute respiratory syndrome coronavirus 2 (SARS-CoV-2) infection by binding to the cell surface receptor for advanced glycation end-products (RAGE). In various diseases, including COVID-19, TOX release was highly detectable in association with disease severity, contributing to lung fibroproliferative acute respiratory distress syndrome (ARDS). Recombinant TOX-induced blood vessel rupture, similar to a clinical signature in patients experiencing a cytokine storm, further exacerbating respiratory function impairment. In contrast, disruption of TOX function by a neutralizing antibody and genetic removal of RAGE diminished TOX-mediated deleterious effects. Altogether, our results suggest an insight into TOX function as an inflammatory mediator and propose the TOX–RAGE axis as a potential target for treating severe patients with pulmonary infection and mitigating lung fibroproliferative ARDS.

## Significance

The study reveals a novel extracellular role of the thymocyte selection–associated high-mobility group box (TOX) protein, identified in response to severe acute respiratory syndrome coronavirus 2 (SARS-CoV-2) infection, elucidating its link to severe inflammatory responses and tissue damage observed in COVID-19 patients. Through inhibition of the TOX–RAGE (receptor for advanced glycation end-products) axis, the research proposes a potential therapeutic target for pulmonary infection–mediated fibroproliferative acute respiratory distress syndrome (ARDS), offering a promising avenue for mitigating cytokine storm, vascular dysfunction, and tissue injury. This groundbreaking insight introduces the TOX protein as a key player in disease severity and offers a valuable strategy for developing targeted therapies, complementing existing medical regimens, to enhance outcomes for critically severe infectious diseases.

Since the first report of the novel severe acute respiratory syndrome coronavirus 2 (SARS-CoV-2) at the end of 2019 (1), it has spread rapidly around the world, causing COVID-19 in humans (2). Clinical analysis revealed that symptoms in patients with COVID-19 were similar to those of other beta-lineage coronaviruses, such as severe acute respiratory syndrome coronavirus (SARS-CoV) and Middle East respiratory syndrome coronavirus (3, 4). Severe SARS-CoV-2 infection in humans mainly causes pneumonia and acute respiratory distress syndrome (ARDS) as well as cytokine release syndrome (CRS) and multiple organ failure (MOF) (5, 6). Severe COVID-19 patients are at a heightened risk due to the potential threat of life-threatening ARDS accompanied by lung fibrosis. Therefore, targeting this condition should be a priority in their treatment.

Histopathological studies of lung tissue from severe COVID-19 cases have revealed distinctive features consistent with ARDS, including diffuse alveolar damage, hyaline membrane formation, and microthrombi in pulmonary vasculature (7). These findings underscore the complexity of lung injury in severe COVID-19, implicating dysregulated immune responses, endothelial dysfunction, and coagulopathy. Immune dysregulation in COVID-19 ARDS is characterized by a hyperinflammatory state, with elevated levels of proinflammatory cytokines such as interleukin-6 (IL-6), tumor necrosis factor-alpha (TNF-α), and interleukin-1β (IL-1β), contributing to pulmonary inflammation and vascular injury (1, 5). Moreover, COVID-19 exhibits systemic manifestations beyond the respiratory system, including cardiovascular complications, renal dysfunction, and neurological sequelae, indicative of widespread endothelial involvement and systemic inflammation (8). Endothelial dysfunction emerges as a central pathophysiological feature of severe COVID-19, facilitating microvascular thrombosis, vascular leakage, and multiorgan dysfunction (9).

Recent studies have shed light on the intricate interplay between viral pathogenesis and host immune responses in driving severe COVID-19 ARDS. Dysregulated type I interferon responses and impaired adaptive immunity have been implicated in disease severity and progression (10, 11). Furthermore, elevated levels of acetylated 676th lysine transforming growth-factor-beta-induced protein (TGFBIp K676Ac) (12), Wnt5a protein (13), and SREBP-2 protein (14) in the blood of patients have been implicated in disease severity of COVID-19 ARDS, highlighting the need for personalized therapeutic approaches.

Current conceptual advances through mapping of SARS-CoV-2 RNA transcriptome (15) and proteomic analysis (16) in the infected cells expand our knowledge and provide fundamental insights to better understand this new coronavirus. Researchers are exploring various medical regimens to efficiently control the continuous spread of SARS-CoV-2 and the COVID-19 pandemic around the world, but high mutagenesis and structural changes in SARS-CoV-2 create barriers to therapeutic development (17, 18).

As of February 29, 2024, the Food and Drug Administration has granted approval for the use of ritonavir-boosted nirmatrelvir, packaged as Paxlovid, in the treatment of mild to moderate COVID-19 in adults who are at high risk of progressing to severe COVID-19. Nirmatrelvir, an oral protease inhibitor, effectively targets the viral protease Mpro, a key player in viral replication responsible for cleaving the two viral polyproteins (19). Its antiviral activity extends to all known coronaviruses that infect humans (20). Paxlovid is a combination therapy that includes nirmatrelvir and ritonavir, the latter serving as a potent cytochrome P450 3A4 inhibitor and a pharmacokinetic boosting agent, commonly used with HIV protease inhibitors. The coadministration of ritonavir is essential to elevate nirmatrelvir concentrations to the desired therapeutic levels. Paxlovid is given to prevent progression to severe diseases.

Thymocyte selection–associated high-mobility group box (TOX) is a transcription factor that belongs to the high-mobility group box (HMG-box) superfamily and has attracted much attention due to its requisite role in the exhaustion of CD8^+^ T cells and specific T cell differentiation during chronic infection and cancer (21–24). Here, we report that a nuclear factor TOX extracellularly promotes inflammatory responses in patients with infectious diseases, including COVID-19, via a receptor for advanced glycation end-products (RAGE). A sepsis model was used to investigate pathogenesis of COVID-19 on account of the connection that both induce systemic proinflammatory immune activation. We first noticed the increase in TOX expression in CD8+ T cells due to inflammatory cytokines (25). In our study, TOX was inadvertently identified during the proteomics analysis of patients’ blood. The released TOX showed a high correlation with disease severity and led to the induction of multiple cytokine, disruption of endothelial barriers, and organ damage. A functional blockade of the TOX–RAGE axis by neutralizing antibodies against TOX or genetic loss of RAGE raised protection against SARS-CoV-2 infection. Our findings not only provide an unrevealed role of TOX as an extracellular mediator in severe inflammation but also suggest the TOX–RAGE axis as a potential therapeutic target for pulmonary infection-mediated fibroproliferative ARDS and septic shock.

## Results

### SARS-CoV-2 Infection Induces TOX Release from PBMCs (Peripheral blood Mononuclear Cell), Which Reflects Disease Severity.

**Fig. 1.**
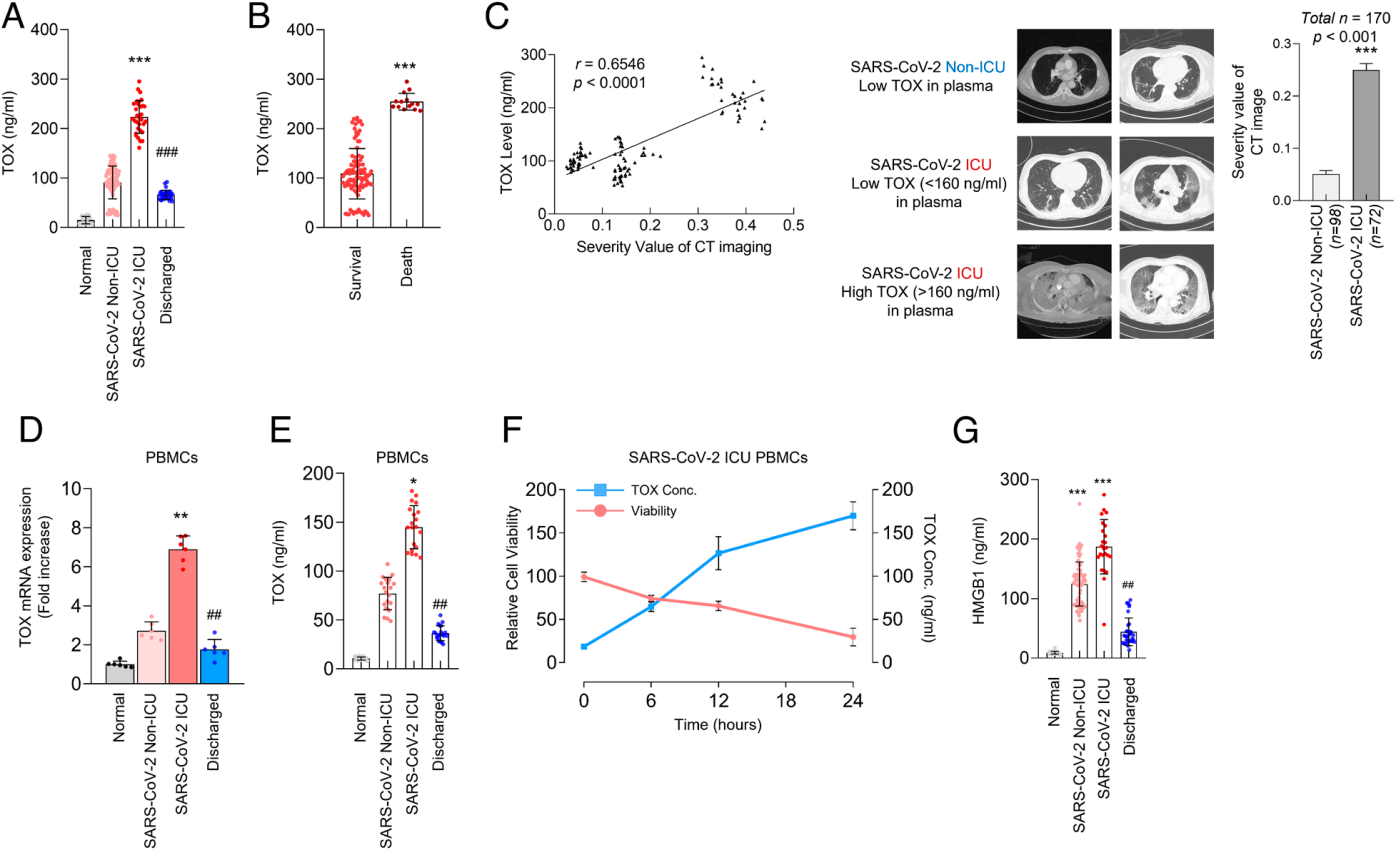
TOX is released upon SARS-CoV-2 infection and its release correlates with disease severity. (*A*) Level of TOX in the blood of SARS-CoV-2 patients depending on the severity of symptoms (***P < 0.001, ^###^*P* < 0.001). (*B*) Level of TOX in the blood of survival and death patients (***P < 0.001). (*C*) CT images of SARS-CoV-2 patients’ lung tissue and severity analysis data using an AI program and severity scoring technique based on the bilateral diffuse ground-glass opacities (GGOs) in the central and peripheral lungs for quantitative assessment of chest CT (26) (n = 170, ***P < 0.001). (*D*) Expression level of TOX mRNA in PBMCs of SARS-CoV-2 pneumonia patients (**P < 0.01, and ^##^*P* < 0.01). (*E*) Level of TOX produced by SARS-CoV-2 patients’ PBMCs (*P < 0.05, and ^##^*P* < 0.01). (*F*) Time-dependent correlation of PBMC viability and TOX level in SARS-CoV-2 ICU patients’ PBMCs. (*G*) HMGB1 levels among COVID-19 patients admitted to the ICU, discharged patients, and healthy controls (***P < 0.001, and ^##^*P* < 0.01). A Student unpaired, two-tailed *t* test was used for comparison of between-group data. Quantification of TOX protein was performed by using ELISA.

To explore the effect of SARS-CoV-2 infection on the cellular and molecular alteration, we first analyzed the blood of patients with different COVID-19 disease severity (*SI Appendix,* Table S1). The total number of white blood cells and neutrophils was significantly increased in the intensive care unit (ICU) than the non-ICU group. Conversely, the number of lymphocytes was reduced around twofold when compared to the non-ICU group. At the molecular level, we observed that hepatic injury markers -alanine aminotransferase (ALT) and aspartate aminotransferase (AST)- and the renal injury markers -blood urea nitrogen (BUN) and creatinine phosphokinase- and the infectious disease marker, C-reactive protein (CRP), were also up-regulated in the ICU group (*SI Appendix,* Table S1). In the process of exploring biomarkers for SARS-CoV-2 infection, we unexpectedly found that a transcription factor TOX was present in the patient’s sera with COVID-19. Through enzyme-linked immunosorbent assay (ELISA) or western blot analysis, we observed that extracellular TOX levels in patients with COVID-19 were much higher than in normal individuals (Fig. 1*A* and *SI Appendix,* Table S1). Further analysis of TOX level according to patient’s severities revealed a significant increase in the ICU than the non-ICU group (Fig. 1*A* and *SI Appendix,* Table S1). Interestingly, the TOX level in the discharged group recovered to a moderate level, which was consistent with its low concentration in the plasma of surviving patients (Fig. 1*B* and *SI Appendix,* Fig. S1). The computed tomography (CT) results clearly showed that COVID-19 patient with high TOX level in plasma suffered severe lung damage compared to those with low TOX level (Fig. 1*C*). Consistently, COVID-19 patients with high TOX levels also exhibited a high level of lactate dehydrogenase (LDH), wildly used as a tissue injury indicator (*SI Appendix,* Fig. S1). Collectively, our findings suggest that a nuclear factor TOX can be released into the sera and can serve as a biomarker indicating patient’s severities with COVID-19.

Next, in order to determine the cellular mechanism for TOX release in human sera with COVID-19, we analyzed PBMCs, known as critical components of the immune system against viral infection. Consistent with observation in COVID-19 patients’ PBMCs derived from SARS-CoV-2-ICU group highly expressed and released TOX when compared to the non-ICU group. Likewise, TOX expression and release in the discharged group returned to a moderate level (Fig. 1 *D* and *E* and *SI Appendix,* Fig. S2). In vitro time-course analysis using SARS-CoV-2 ICU patient-derived PBMCs further confirmed that the cell viability decreased as the TOX extracellularly increases (Fig. 1*F*). SARS-CoV-2 causes increase in susceptibility to other diseases following infection (27); in fact, patients with pneumonia or sepsis exhibit similar TOX release to COVID-19 patients with the same symptoms (*SI Appendix,* Fig. S3). To identify the main cellular source of TOX, we measured the TOX release from Pan T cells, monocytes, and neutrophils using ELISA. The results demonstrated that T cells are involved in the release of TOX during infection (*SI Appendix,* Fig. S3). Furthermore, immune suppression was detected by the release of soluble inhibitory immune checkpoint proteins sCTLA-4, sPD-1, and sPD-L1 (*SI Appendix,* Fig. S3). The supportive data that levels of soluble immune checkpoints in the sera of COVID-19 patients explain, although indirectly, high and sustained TOX is associated with immune exhaustion. These results suggested that TOX released from PBMCs can act as an endotoxin mediator to induce proinflammatory responses. Limulus amebocyte lysate (LAL) assay was performed for the presence of endotoxins; however, no endotoxin was detected in the recombinant TOX (rTOX), demonstrating that the rTOX used in the experiment was free from endotoxin contamination (*SI Appendix,* Fig. S4*A*). As expected, addition of rTOX to normal PBMCs was sufficient to activate NF-κB (nuclear factor kappa B) signaling in the dose-dependent manner, demonstrating that the effect is solely due to TOX signaling (*SI Appendix*, Fig. S4*B*). Additionally, SARS-CoV-2 caused infected cells to release HMGB1, late inflammatory mediator, which exacerbated the severe inflammatory responses and MOF (Fig. 1*G*). Overall, our results suggest that TOX can be released in response to inflammation and can act as an indicator determining disease severity.

### Functional Blockade of TOX by Neutralizing Antibody Prevents Induction of Cytokine Release.

**Fig. 2.**
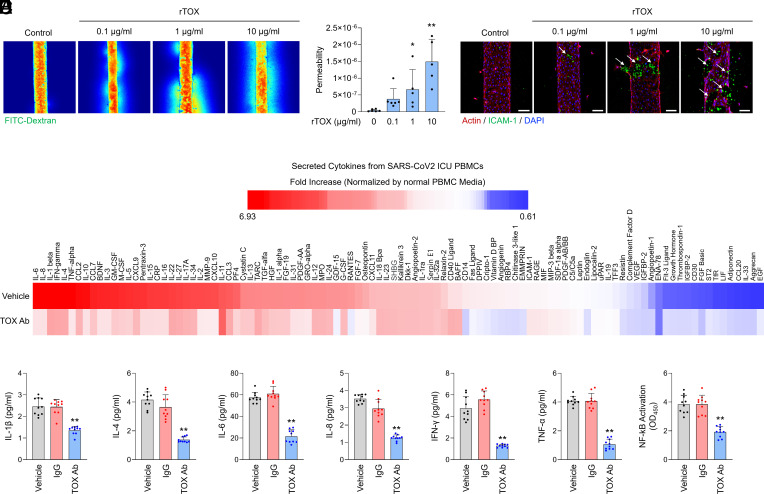
TOX is associated with cytokine storm, inflammation, and endothelial barrier disruption, and pharmacological inhibition of TOX suppresses the cytokine storm. (*A*) Visualization of TOX-mediated vascular damage using engineered blood vessel chip. The concentration of exogenous TOX (rTOX) administration was varied to observe the degree of leakage of the fluorescent material (40 kDa FITC-conjugated dextran). (*B*) Quantified transendothelial permeability from *A* (*P < 0.05 and ***P < 0.001). (*C*) Visualization of vascular inflammation and damage by immunofluorescence staining. ICAM-1 (green), F-actin (red), and nucleus (DAPI). Localization of the vascular damage at high inflammation sites is indicated (white arrows). (*D*) Effect of pharmacological TOX inhibition using TOX antibody. Cytokine array analysis by addition of exogenous TOX antibody to SARS-CoV-2 ICU patients’ PBMCs. (*E*) IL-1β, IL-4, IL-6, IL-8, IFN-γ, TNF-α by addition of exogenous TOX antibody to SARS-CoV-2 ICU patients’ PBMCs using ELISA. (*F*) Suppression of NF-κB signaling activation by exogenous addition of TOX antibody to SARS-CoV-2 ICU patients’ PBMCs (**P < 0.01). A Student unpaired, two-tailed *t* test was used for comparison of between-group data.

Recent report suggests that the severity of COVID-19 is linked to blood vessel damage, leading to widespread thrombosis, or blood clotting, in the lung. As our findings have shown that extracellular TOX may mediate inflammatory responses of SARS-CoV-2 infection, we examined the effect of TOX on the deterioration of blood vessels in a 3D chip environment. The perfusable 3D in vitro blood endothelium was prepared by culturing human umbilical vein endothelial cells (HUVECs) in the collagen gel to test the vascular damage induced by TOX. When the TOX was introduced into the engineered blood vessel, both the vascular leakage and the permeability were markedly increased in a dose-dependent manner (Fig. 2 *A* and *B*). Immunostaining analysis further showed that the vascular damage (shown as a reduced actin staining which is a whole hallmark of disrupted vascular structure) was predominantly located at the site of inflammation when determined by an inflammation marker, intercellular adhesion molecule 1 (ICAM-1) (Fig. 2*C*). Consistently, TOX-induced dose-dependent ICAM-1 expression as well as vasculature rupture. TOX-mediated vascular damage speculated the possibility that various endotoxic proteins released from the vasculature could cause MOF in the body.

Our findings demonstrate that TOX treatment is associated with inflammatory responses and subsequent vascular damage led us to further investigate whether extracellular inhibition of TOX can attenuate multiple cytokine induction caused by SARS-CoV-2 infection. To do this, we first profiled the differential levels of cytokines. Many proinflammatory cytokines such as IL-1β, IL-6, and TNF-α were more released by PBMCs of SARS-CoV-2 ICU, while some cytokines, including IL-33 and CCL20, were down-regulated (Fig. 2 *D*, *Upper* lane). Importantly, treating TOX-neutralizing antibodies in PBMCs isolated from SARS-CoV-2-ICU dramatically prohibited excessive production of cytokines (Fig. 2 *D*, *Bottom* lane). It was further evaluated that extracellular TOX inhibition significantly inhibits preinflammatory cytokine induction, NF-kB signaling, and cell viability in PBMCs. (Fig. 2 *E* and *F* and *SI Appendix,* Fig. S5). These results suggest that the TOX-neutralizing antibody can be a valuable therapeutic agent inhibiting cytokine storm for COVID-19 patients.

### RAGE Is a Receptor and Mediates TOX-Induced Severe Inflammatory Responses.

**Fig. 3.**
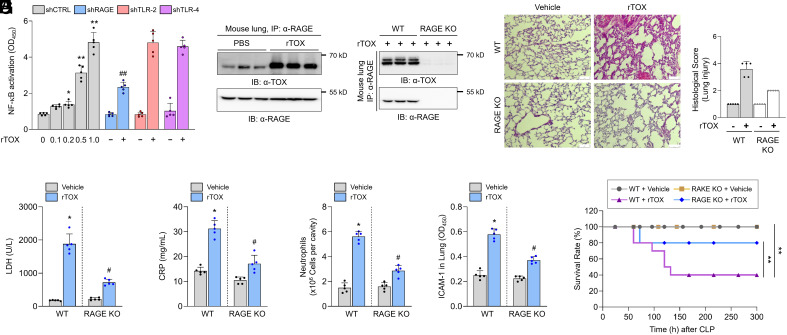
RAGE acts as a receptor for extracellular TOX (rTOX). (*A*) Effect of RAGE, TLR-2, and TLR-4 knockdown in the NF-κB signaling activation by introduction of rTOX in vitro (**P < 0.01 and ##*P* < 0.01). (*B* and *C*) rTOX intravenously injected mice were killed, and isolated lung tissue lysates were immunoprecipitated. Detection of TOX or RAGE with anti-TOX or anti-RAGE antibodies after IP with anti-RAGE antibody in *B* WT mouse lung or (*C*) RAGE-KO mouse lung for TOX–RAGE interaction. (*D*) Histology and histological score of mouse lung tissue 72 h after the intravenous injection of 0.1 mg/kg TOX. Comparison of lung failure in WT 0.1 mg/kg TOX (*Right Top*) and the KO of RAGE (*Right Bottom*). (Scale bar: 75 μm.) (*E*–*H*) Effect of RAGE KO in (*E*) LDH, (*F*) CRP, (*G*) number of neutrophils, and (*H*) ICAM-1 expression in lung tissue. (*I*) Effect of RAGE KO in the survival rate of the mouse (**P < 0.01). TOX was delivered via intravenous injection with a concentration of 0.1 mg/kg. A Student unpaired, two-tailed *t* test was used for comparison of between-group data.

To find molecular mechanisms by which extracellular TOX induces inflammatory response and subsequent endothelial barrier disruption, we first speculated TOX can act as a ligand for an inflammatory receptor. To identify a receptor candidate for the TOX ligand, we focused on the HMGB1 nuclear factor with the HMG-box domain that performs dual functions, transcription factor and extracellular ligand, in which TOX behaves. HMGB1 can be released by immune cells or damaged cells, and trigger inflammation via binding multiple receptors such as RAGE, TLR-2, or TLR-4. Therefore, we first tested whether TOX can also act via these inflammatory receptors and found that shRNA-mediated deletion of RAGE, but not TLR-2 or TLR-4, in HUVECs significantly repressed NF-kB activation and vascular permeability induced by rTOX treatment, indicating that RAGE may act as an inflammatory receptor for TOX ligand (Fig. 3*A* and *SI Appendix,* Fig. S6*A*). Therefore, we performed immunoprecipitation (IP) assay to test whether TOX binds RAGE. Protein extracts from HUVEC or mouse lung were incubated with rTOX, and subjected to IP with anti-RAGE antibody (Fig. 3*B* and *SI Appendix,* Fig. S6*B*). Indeed, we observed the association between TOX and RAGE. TOX–RAGE interaction was further corroborated because it was undetectable in RAGE-depleted HUVECs or RAGE-knockout (KO) mouse lungs (Fig. 3*C* and *SI Appendix,* Fig. S6*C*).

### TOX Induces Lung Damage in the Mouse Model in RAGE-Dependent Manner.

Most patients with SARS-CoV-2 infection suffered from ARDS and MOF, including lung and kidney. In particular, since the lungs have known to be a primary target for SARS-CoV-2 infection and we showed that upregulation of TOX release from PBMCs collected from severe COVID-19 patients induces inflammatory responses and vascular leakage, we first investigated whether TOX is sufficient to damage lung tissue. Mice that were intravenously (i.v.) injection with rTOX dose-dependently suffered from severe lung damage, as evidenced by its histology (*SI Appendix,* Fig. S7*A*). Importantly, RAGE-KO mice were dramatically resistant to TOX-mediated lung tissue dysfunction (Fig. 3*D*). Consistent with these gross tissue abnormalities, tissue damage-related markers, including ALT, AST, BUN, LDH, creatinine, and CRP, were robustly induced in rTOX-injected mice, while genetic removal of RAGE significantly suppressed them (Fig. 3 *E* and *F* and *SI Appendix,* Fig. S7 *B*–*E*). Likewise, the permeability of vasculature, the number of neutrophils, and ICAM-1 expression were highly increased in rTOX-injected mice. However, these cellular and molecular indicators for hyperinflammatory response were significantly reduced in RAGE-KO mice (Fig. 3 *G* and *H* and *SI Appendix,* Figs. S7 and S8). The combination of these effects resulted an earlier death of the rTOX-injected mice compared with normal mice, but genetic loss of RAGE significantly prolonged survival (Fig. 3*I*). When TOX antibody was administered, increase in the survival rate, reduction in lung tissue damage and tissue damage markers in septic mice model were demonstrated (*SI Appendix*, Figs. S9 and S10). Through in vitro and in vivo analyses, our results demonstrated that high TOX release axis mediates hyperinflammatory response to induce organ dysfunction, eventually inducing severe CRS and sepsis.

### TOX-Induced Fibroproliferative ARDS in Mouse.

**Fig. 4.**
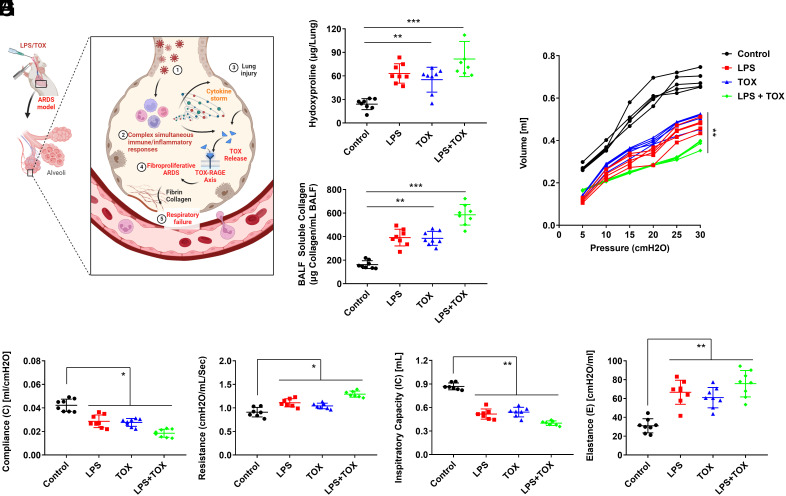
TOX induces impairment of respiratory functions. (*A*) The mechanistic role of the TOX–RAGE axis in inducing systemic severe inflammation: Respiratory virus infection triggers complex simultaneous immune/inflammatory responses, leading to cytokine storm and lung damage. TOX is subsequently released and binds to the RAGE receptor. The TOX–RAGE axis induces fibroproliferative ARDS, resulting in respiratory failure. (*B* and *C*) Effect of intratracheal injection of rTOX on (*B*) lung tissue hydroxyproline levels and (*C*) collagen release in BALF. (*D*–*H*) Respiratory function is indicated by (*D*) PV curve, (*E*) lung compliance, (*F*) resistance, (*G*) lung IC, and (*H*) elastance. *P < 0.05, **P < 0.01 and ***P < 0.001. A Student unpaired, two-tailed *t* test was used for comparison of between-group data.

In the above results, we confirmed the role of the TOX–RAGE axis in inducing systemic severe inflammation and aimed to validate the mechanism by which TOX protein, released by respiratory virus infections such as SARS-CoV-2, contributes to pulmonary damage (Fig. 4*A*). Intratracheal injections of PBS, lipopolysaccharide (LPS), rTOX, and LPS + rTOX were administered, and after 7 d, the group treated with rTOX exhibited a significant increase in the lung tissue hydroxyproline levels, compared to the group treated with LPS (Fig. 4*B*). Additionally, collagen release in bronchoalveolar lavage fluid (BALF) showed an increase in the rTOX-administered group (Fig. 4*C*). Evaluation of respiratory function in each group of mice revealed notable findings. Pressure–volume (PV) curves, assessed using the FlexiVent FX system (SCIREQ Inc. Montreal, Canada), exhibited a right-downward shift in LPS and rTOX-challenged mice, indicative of decreased lung compliance compared to PBS-administered mice (Fig. 4 *D* and *E*). In the rTOX-administered group, PV curves exhibited a significant reduction, along with decreased lung compliance and inspiratory capacity (IC), as well as increased resistance and elastance compared to the group treated with LPS alone (Fig. 4 *D*–*H*). We then validated the lung epithelial cell damage in mice by trichrome and α-SMA staining for fibrosis parameter assessment, and terminal deoxynucleotidyl transferase biotin-dUTP nick end labeling (TUNEL) staining for apoptosis. In the rTOX-administered group, trichrome, α-SMA, and apoptosis were observed, demonstrating that fibrosis and cell death had occurred at the damaged site (*SI Appendix*, Figs. S11 and S12). Furthermore, a synergistic increase in fibroproliferative ARDS was observed when LPS and rTOX were administered concurrently (Fig. 4). These findings validate the role of TOX in inducing inflammatory responses and fibrosis, ultimately contributing to respiratory impairment.

## Discussion

TOX was originally identified as a highly up-regulated transcription factor in CD4^+^/CD8^+^ double positive thymocytes during thymic selection events and is known to play pivotal roles in the immune system, including T cell and NK cell development (28–31). TOX-deficient mice show severe defect in the development of T cell subsets (22). More recent studies suggest that TOX promotes the exhausted T cells phenotype upon the sustained antigen stimulation (22, 32, 33). However, so far, these functions of TOX in the immune system depend on its role as a transcription activator. In this study, we report the unrevealed function of TOX as an extracellular ligand, not a nuclear factor, in response to SARS-CoV-2 infection. TOX was present in COVID-19 patient sera and its high release was associated with multiple cytokine inductions and organ failure, highlighting disease severity. A possible reason for the elevation of TOX level in sera of ICU groups could be that because the total number of lymphocytes remained low, the proportion of exhausted T cells in the ICU group may have increased. Liu et al. reported a similar finding where a decrease in the number of lymphocytes in the early stages in critically ill patients was observed (34). As the vascular integrity is damaged by infection, lymphocytes in the blood may get concentrated at the site of infection due to the decrease in the number of lymphocytes in the blood. In patients with severe inflammation, the decrease may be more severe as lymphocytes escape from the blood due to vascular leakage. Notably, the engineered vasculature and in vivo model system further evaluated that extracellular TOX-induced inflammatory response and tissue injury, which could be reversed in RAGE-deficient HUVEC or mice. These results suggest that TOX released in SARS-CoV-2 infection may depend on the RAGE receptor.

ARDS is a condition characterized by widespread inflammatory lung damage, accompanied by escalated pulmonary edema and the swift onset of hypoxemic respiratory failure. Despite advancements, ARDS remains inadequately addressed, marked by elevated mortality rates and a limited array of efficacious treatments. Within the lung, it has been reported that the membrane RAGE is prominently expressed in alveolar type-1 epithelial cells (35), and activation of RAGE serves as an indicator of epithelial injury (36). The RAGE ligands induce lung injury by activating the RAGE-dependent signaling pathways. While in idiopathic pulmonary fibrosis, an increased ratio of AGE/RAGE was observed (37). The fibrotic lung is associated with an AGE/RAGE imbalance, which is believed to induce oxidative damage to the lung.

RAGE is known to be triggered by a variety of ligands and activates several signaling pathways. Binding of RAGE ligands, such as S100 proteins, AGE, and HMGB1 to V-domain of RAGE on the surface of type 2 alveolar epithelial cells lead to activation of proinflammatory transcription factors, activator protein 1, signal transducer and activator of transcription three, and NF-κB. As a consequence, cytokines such as interleukins (IL-1, IL-6, IL-8, IL-10) and TNF-α are elevated (12, 38). Similar to HMGB1, it is considered that TOX may also activate the RAGE-dependent pathways, resulting in a massive cytokine storm.

The TOX’s dual function was similar to that of HMGB1, which also belongs to HMG-box family and exerts various biological functions both inside and outside the cell. In the extracellular space, HMGB1 functions as a damage-associated molecular pattern by passively releasing from a dying or injured cell or immune cell upon infection. HMGB1 acts as a proinflammatory mediator by directly binding to the receptor for RAGE, TLR-2, and TLR-4. Previously, persistent elevation of HMGB1 in patients with severe sepsis or septic shock was observed (39). However, HMGB1 levels in these patient’s sera were not comparably correlated with severity of infection or cytokine induction. On the other hand, our findings described in this study showed a significant correlation between TOX and diverse inflammatory indicators upon infection, suggesting that TOX may be a superior candidate as a biomarker compared to other HMGB1 family proteins. It has been recently reported that HMGB1–LPS complex was internalized via RAGE-mediated endocytosis, where HMGB1 facilitated LPS translocation from endolysosomes to the cytosol by inducing lysosomal rupture (40, 41). Likewise, it is speculated that endotoxin such as TOX may also internalize via RAGE-mediated endocytosis and subsequently activate caspase-dependent pyroptosis, thereby inducing lethality associated inflammatory mediators (40). TOX protein only has RAGE binding domain, so it is speculated that TOX may act by binding to RAGE rather than TLR-4. In addition, regarding to extracellular function of TOX, it would be intriguing to explore further the molecular mechanisms on how TOX is released from the cell. In this study, we utilized a sepsis mouse model to show reduction in the symptoms using anti-TOX antibodies, which while relevant in severe COVID-19, is not entirely a substitute for a natural infection model. The connection between sepsis as the model and COVID-19 as a disease is that both induce systemic proinflammatory immune activation. Thus, incorporating a COVID-19 infection model into our future research may substantially support our findings.

We provide several lines of evidence that TOX was highly released upon SARS-CoV-2 infection, and cells or tissues exposed to TOX can be damaged. Indeed, inhibition of TOX-RAGE association via treatment of TOX antibody efficiently protected cells or organs from cytokine storm, vascular dysfunction, and tissue injury (*SI Appendix,* Figs. S9 and S10). Therefore, the TOX–RAGE axis could be a potential therapeutic target for pulmonary infection-mediated fibroproliferative ARDS. In addition, strategies targeting the TOX–RAGE axis can also be combined with other medical regimens such as remdesivir, which may lead to great efficacy in improving patient outcomes.

## Materials and Methods

### Patient and Normal Serum Samples.

The patient was admitted to the department of internal medicine, Yeungnam University Medical Center, after SARS-CoV-2 infection was confirmed at a public health center in Daegu. SAR-CoV-2 ICU is a group that has progressed to ARDS or sepsis and receives respiratory intensive care in ICU. Healthy volunteers were used as controls and clinical data of all patients were collected. Plasma samples were generated by centrifuge at 2,000 × g for 5 min within 48 h after whole blood collection. The study protocol (YUH 2018-05-022, 2020-03-057, 2020-05-031-001) was approved by the Institutional Review Board of Yeungnam University Hospital at Daegu in Korea. These experiments were carried out with the full, informed consent of the subjects.

### CT Analysis.

We adopted a clinical significance verified AI program for determination of the severity from CT images of patients who donated blood samples (n = 170) (42, 43). We established the CT analysis technique based on the severity with a value difference of 8.

### ELISA for TOX and HMGB1.

We performed competitive ELISA using antibodies from Cell Signaling Technology that recognize human TOX (catalog number 99036) or antibodies from Invitrogen that recognize human HMGB1 (catalog number PA1-16926). The high purity rTOX protein used in our experiments was obtained from Abcam (catalog number ab160644) and is a full-length protein with 526 amino acids (UniProtKB accession number: NP_055544). rTOX (1 μg/100 μL dilution) or plasmas were coated onto Nunc-Immuno™ MicroWell™ 96 well plates and incubated overnight at 4 °C. Prior to use, the plates were washed three times with phosphate-buffered saline with Tween 20 (PBST) and blocked with 3% bovine serum albumin (BSA) in PBS for 30 min 37 °C. Primary antibody (1:2,000 dilution) and plasma sample (20 μg) were preincubated for 1 h at 37 °C, and then, the preincubated sample was transferred to peptide-coated plate and incubated for 1 h at 37 °C. The plate was washed five times with PBST and then further incubated with horseradish peroxidase-conjugated (1:5,000 dilution) for 30 min at 37 °C. The washed plate was treated with TMB ELISA substrate 100 μL/well for 10 min 37 °C, and then, Stop Solution 100 μL/well was added. The detection was performed at 450 nm by microplate reader (TECAN).

### Western Blotting and IP.

TOX in plasma of healthy volunteers, SARS-CoV-2 non-ICU patients, SARS-CoV-2 ICU patients were detected by immunoblotting. After sodium dodecyl sulfate–polyacrylamide gel electrophoresis (SDS-PAGE), we performed an immunoblotting assay with each antibody. Plasmas (20 μg) were incubated with the anti-TOX antibody (catalog number 99036, Cell Signaling Technology) or anti-transferrin antibody (catalog number ab109503) at 4 °C overnight. For IP experiments, the immunoprecipitants were recovered using anti-RAGE antibody-bound protein A/G-S magnetic beads, washed four times with IP buffer, and resuspended in the sample buffer of SDS-PAGE, followed by boiling for 10 min. Bound proteins were then analyzed by immunoblotting using anti-TOX or anti-RAGE antibodies.

### PBMCs Isolation.

PBMCs were isolated on a Percoll (pH 8.5 to 9.5; Sigma-Aldrich, UK) density gradient as previously described (44). The PBMCs (95% purity and 97% viable according to trypan blue exclusion) were resuspended in RPMI 1640 media (Sigma-Aldrich).

### Real-Time PCR of TOX.

To generate complementary DNA (cDNA) from PBMCs, 1 μg of total RNA was reverse transcribed with random hexamers using expand RTP (Roche). Real-time PCR was performed using the LightCycler FastStart DNA Master SYBR Green I from Roche Diagnostics GmbH according to the manufacturer’s protocol. The following LightCycler conditions were used: initial denaturation at 95 °C for 10 min, followed by 45 cycles with denaturation at 95 °C for 10 min, annealing at 60 °C for 5 min, and elongation at 72 °C for 15 min. Quantities of specific mRNA in the sample were measured according to the corresponding gene-specific standard curves.

### WST-1 Cell Proliferation Assay.

10 μL per well of water-soluble tetrazolium 1 (WST-1) reagent were added in PBMC cultured with/without TOX antibody and incubated at 37 °C with 5% CO_2_. At indicated time points, measurements of absorbance were taken at 480 nm and 600 nm (background) on Tecan Spark microplate reader.

### NF-κB Transcriptional Activity Assay.

Nuclear extracts preparation and TransAM assay were performed as previously described (12, 45). The activity of individual NF-κB subunits was detected by an ELISA-based NF-κB family transcription factor assay kit (43296; Active Motif, Carlsbad, CA, USA). Briefly, nuclear extracts (2 µg) were incubated in a 96-well plate, which were immobilized NF-κB consensus oligonucleotides. The captured complexes were incubated with specific NF-κB primary Abs and subsequently detected with horseradish peroxidase (HRP)-conjugated secondary Abs (included with the kit). Finally, the optical density (OD) value at 450 nm was measured by Tecan Spark microplate reader (Tecan, Austria GmbH, Austria).

### Plasma Cytokine Profiling.

Plasma pools of patients with normal or SARS-CoV-2 patients were processed as indicated in the Human XL Cytokine Array Kit (R&D Systems). Developed films were scanned, and the obtained images were analyzed using ImageJ version 1.43.

### Animals and Husbandry.

RAGE KO mice were constructed on an SVE129×C57BL/6 background (129/B6) as previously described (26) and were backcrossed >six generations into C57BL/6. C57BL/6 male mice (6- to 7-wk-old, weighing 18 to 20 g) were obtained from Orient Bio (Seongnam, Korea) and used after a 12 d acclimatization period. Five animals per cage were housed under controlled temperature at 20 to 25 °C and humidity of 40 to 45% with a 12:12 h light/dark cycle. Mice were fed a normal rodent pellet diet and supplied with water ad libitum. All experiments were performed according to institutional animal care and use committee–approved protocols.

### ELISA for IL-1β, IL-4, IL-6, IL-10, IFN-γ, and TNF-α.

The concentrations of cytokines in SARS-CoV-2 patients’ plasma were quantified according to the manufacturer’s instructions using a commercially available ELISA kit. Values were measured using an ELISA plate reader (Tecan, Austria GmbH, Austria). Human IL-1β Quantikine ELISA Kit (DLB50, R&D Systems, Minneapolis, MN), Human IL-4 Quantikine ELISA Kit (D4050, R&D Systems, Minneapolis, MN), Human IL-6 Quantikine ELISA Kit (D6050, R&D Systems, Minneapolis, MN) Human IL-10 Quantikine ELISA Kit (D1000B, R&D Systems, Minneapolis, MN), Human IFN-γ Quantikine ELISA Kit (DIF50, R&D Systems, Minneapolis, MN), and Human TNF-α Quantikine ELISA Kit (DTA00D, R&D Systems, Minneapolis, MN).

### In Vitro Permeability Assay.

To quantify changes in endothelial cell permeability in response to increasing concentrations of each construct, we employed a modified two-compartment chamber model as previously described (46). HUVECs were seeded at a density of 5 × 10^4^ cells per well in 12-mm-diameter Transwells with a pore size of 3 µm and cultured for 3 d. Upon reaching confluency, HUVEC monolayers were treated with rTOX at concentrations up to 1 μg/mL for 12 h. Following treatment, Transwell inserts were rinsed with PBS (pH 7.4) and exposed to Evans blue dye solution (0.5 mL; 0.67 mg/mL) diluted in growth medium containing 4% BSA. Fresh growth medium was added to the lower chamber, while the upper chamber received medium supplemented with Evans blue dye and BSA. After a 10-min incubation period, the OD of the sample in the lower chamber was measured at 650 nm.

### Fabrication of Blood Vessel on a Chip.

As a housing that surrounds cell-laden type I collagen, polydimethylsiloxane (PDMS, Dow corning, Inc.) was used. To use microneedles as the template for channel formation, a metal frame which has uniformly spaced holes was prepared. Sylgard 184 PDMS prepolymer was mixed with a curing agent in the ratio of 10:1 and poured onto the mold in which three microneedles (DASAN Cut, Korea) are readily inserted. After the removal of air bubbles, the PDMS mixture was polymerized at 80 °C for 1 h. After manually removing the needles, the 1st layer was punched in a rectangular shape (4 mm × 10 mm) and then bonded to the 2nd flat PDMS layer with a thickness of 1 mm by oxygen plasma treatment (FEMTO Science, Korea). The circular holes (reservoir: 8 mm, collagen injection port: 1 mm) were also punched in the integrated PDMS layers using the biopsy punches (Miltex, USA) to make reservoirs. The microneedles were injected into the PDMS channel again and then bonded to the slideglass after the oxygen plasma treatment. The neutralized collagen solution was injected in the rectangular chamber and incubated for 30 min for gelation. By removing microneedles from the gelled collagen, microchannels having identical dimensions with microneedles are formed. As an endothelial cell, HUVEC was purchased from Lonza (Basel, Switzerland) and cultured in the recommended culture medium according to the protocol supplied by the manufacturer. For the seeding of HUVECs, a suspension of HUVECs (5 × 10^6^ cells/mL) was loaded into the collagen microchannels, and then, the device was placed in the incubator for 10 min. To increase areas of initial cell adhesion, the device was quickly flipped over and incubated for another 10 min. Cells were maintained in a humidified atmosphere with 5% CO_2_ at 37 °C.

### Permeability Assay in a Blood Vessel on a Chip.

To measure transendothelial permeability across our microfluidic microvasculature, 10 μM of 40 kDa fluorescein isothiocyanate (FITC)-dextran (Sigma-Aldrich) in PBS was used. Immediately after filling collagen microchannels with the FITC-dextran solution, the flow was stopped to allow for transient interfacial diffusion with an instantaneous initial condition. The temporal evolution of molecular transport was acquired by capturing sequential fluorescence images for an initial 5 min with the Zeiss LSM700 laser scanning confocal microscope (Zeiss). The acquired images were color-mapped and mean fluorescence intensity values across microchannels were analyzed with custom-written MATLAB (MathWorks) codes. Then, temporal profiles of the mean fluorescence intensity from edges of the microchannel were fitted with our analytical model to estimate the transendothelial permeability.

### Survival Rate of rTOX Administration.

The mice were i.V. or intratracheal injected with 0.05 or 0.1 mg/kg rTOX and 3 mg/kg LPS. Animal experiments were performed according to the protocols approved by Animal Experimentation Ethics Committee in the Animal Care Committee.

### Hematoxylin and Eosin Staining.

Wild-type (WT) or RAGE-KO C57BL/6 mice were i.V. administered rTOX at doses of 50 or 100 μg/kg. The mice were killed 48 h postinjection, and lung samples were collected, washed thrice with PBS (pH 7.4) to remove residual blood, and fixed in 4% formaldehyde solution (Junsei, Tokyo, Japan) in PBS (pH 7.4) for 20 h at 4 °C. Following fixation, samples underwent dehydration using an ethanol series, embedding in paraffin, sectioning into 4μm-thick slices, and mounting onto slides. Deparaffinization was carried out in a 60 °C oven followed by rehydration and staining with hematoxylin (Sigma). To mitigate overstaining, slides were briefly immersed three times in 0.3% acid alcohol and counterstained with eosin (Sigma). Subsequently, slides were washed in an ethanol series and xylene before being covered with coverslips. Evaluation of lung inflammation was conducted using a previously described histological scoring system (12), where Scores 1 through 4 corresponded to varying degrees of infiltration, alveolar thickening, and peribronchial changes.

WT or RAGE KO C57BL/6 mice were i.V. injected with rTOX (50 or 100 μg/kg). The mice were killed at 48 h after injection. To analyze phenotypic changes in the lungs of TOX-injected mice, lung samples were obtained from each mouse, washed three times with PBS (pH 7.4) to remove residual blood, and fixed in 4% formaldehyde solution (Junsei, Tokyo, Japan) in PBS (pH 7.4) for 20 h at 4 °C. After fixation, the samples were dehydrated using an ethanol series, embedded in paraffin, sectioned into 4 μm-thick sections, and placed on a slide. The slides were deparaffinized in a 60 °C oven, rehydrated, and stained with hematoxylin (Sigma). To remove overstaining, the slides were rapidly dipped three times in 0.3% acid alcohol and were counterstained with eosin (Sigma). The slides were then washed in an ethanol series and xylene and were covered with a coverslip. Histological scoring system was used for the evaluation of lung inflammation as described previously (12). Briefly, Score 1 is characterized by the presence of mild infiltration, alveolar thickening, and mild peribronchial infiltration. Score 2 is characterized by moderate infiltration, alveolar thickening, and moderate peribronchial infiltration. Score 3 is characterized by severe infiltration, alveolar thickening, and severe peribronchial infiltration. Score 4 is characterized by diffusion and very severe peribronchial infiltration.

### In Vivo Permeability Assays.

WT or RAGE KO C57BL/6 mice were i.v. injected with rTOX, followed by an additional injection of 1% Evans blue dye solution in normal saline after 48 h. After a 30 min incubation period, mice were euthanized, and peritoneal exudates were collected following a saline wash (5 mL) and subsequent centrifugation at 200 × g for 10 min. Absorbance of the supernatant was measured at 650 nm. Vascular permeability was quantified based on the amount of dye (μg/mouse) that translocated into the peritoneal cavity, as determined by referencing a previously established standard curve for Evans blue dye (46).

### In Vivo Neutrophils Migration Assays.

For the assessment of neutrophil migration, WT or RAGE KO C57BL/6 mice were i.V. injected with rTOX. After 48 h, mice were killed, and the peritoneal cavities were washed with 5 mL of normal saline. The number of neutrophils was counted using an auto hematology analyzer (Mindray, BC-5000 Vet). The results are expressed as neutrophils × 10^6^ per peritoneal cavity.

### ICAM-1 Expression in the Lung.

Expression of ICAM-1 in the lung was determined by performing direct ELISA. Isolated lung lysates coated onto Nunc-Immuno™ MicroWell™ 96 well plates and incubated overnight at 4 °C. Prior to use, the plates were washed three times with PBST and blocked with 3% BSA in PBS for 30 min 37 °C. Anti-ICAM-1 antibody (1:1,000 dilution) incubated for 1 h at 37 °C. The plate was washed five times with PBST. Secondary antibody (1:5,000 dilution) was incubated for 30 min at 37 °C, and then, the plate was washed five times with PBST. The washed plate was treated with TMB ELISA substrate 100 μL/well for 10 min 37 °C and then Stop Solution 100 μL/well was added. The detection was performed at 450 nm by microplate reader (TECAN).

### Cytokine Levels in the Plasma of Septic Mice.

Fresh serum was used for analysis of AST, ALT, BUN, creatinine, and LDH levels using biochemical kits (MyBioSource). Values were measured using an ELISA plate reader (Tecan, Austria GmbH, Austria).

### Blood Cells Count.

The number of blood cells was counted using an auto hematology analyzer (Mindray, BC-5000 Vet) at the Chiral Material Analysis Center of Sungkyunkwan University.

### Cecal Ligation and Puncture (CLP).

For induction of sepsis, male mice were anesthetized with 2% isoflurane (Forane, JW pharmaceutical, South Korea) in oxygen delivered via a small rodent gas anesthesia machine (RC2, Vetequip), first in a breathing chamber and then via a facemask. They were allowed to breathe spontaneously during the procedure. The CLP-induced sepsis model was prepared as previously described (43). In brief, a 2cm midline incision was made to expose the cecum and adjoining intestine. The cecum was then tightly ligated with a 3.0-silk suture at 5.0 mm from the cecal tip and punctured once using a 22-gauge needle for induction of high-grade sepsis. It was then gently squeezed to extrude a small amount of feces from the perforation site and returned to the peritoneal cavity. The laparotomy site was then sutured with 4.0-silk. In sham control animals, the cecum was exposed but not ligated or punctured and then returned to the abdominal cavity.

### Hydroxyproline and Collagen Assay.

Mice in the respective groups were subjected to intratracheal injection of the following treatments: LPS (5 mg/kg), TOX (0.1 µg/kg), and a combination of LPS and TOX, as appropriate. At the end of the experimental period (48 h), the mice were euthanized, and lung tissues or BALF were carefully excised and collected for further analysis. Hydroxyproline levels in lung tissues were determined using a commercial kit (MAK008, Sigma-Aldrich) in accordance with the manufacturer’s instructions. The quantification of total lung collagen present in the BALF was carried out using the Sircol collagen assay kit (Biocolor Ltd., Newtownabbey, UK), following the manufacturer’s recommended protocol.

### Immunohistochemistry.

Mice in the respective groups were subjected to intratracheal injection of the following treatments: LPS (5 mg/kg), TOX (0.1 mg/kg), and a combination of LPS and TOX, as appropriate. At the end of the experimental period (168 h, 7 d), the mice were euthanized, and lung tissues were carefully excised and collected for further analysis. Lung slides (10 μm thickness) were used for detecting lung fibrosis. The slides were fixed in 4% paraformaldehyde solution (Biodsesang, Korea) in PBS, (pH 7.4). Permeabilization was performed with 0.05% Triton X-100 in PBS 10 min. After permeabilization, slides were incubated with rabbit anti-collagen 1(ab34710, Abcam) or mouse anti-α-smooth muscle actin (ab7817, Abcam) overnight at 4 °C. Subsequently, slides were incubated with anti-rabbit Alexa Fluor 488 (A11034, Invitrogen) or anti-mouse Alexa Fluor 594 (A11032, Invitrogen) secondary antibody for 1 h at room temperature. After PBS-T washing, the slides were mounted with fluorescence medium (Vector Laboratories) and analyzed using a fluorescence microscope (Leica Microsystem, Germany).

### Masson’s Trichrome Staining.

Mice in the respective groups were subjected to intratracheal injection of the following treatments: LPS (5 mg/kg), TOX (0.1 mg/kg), as appropriate. At the end of the experimental period (168 h, 7 d), the mice were euthanized, and lung tissues were carefully excised and collected from each mouse, washed three times in PBS (pH 7.4) to remove remaining blood, and fixed in 4% paraformaldehyde solution (Biosesang, Korea) in PBS (pH 7.4) for 1 h at 4 °C. After fixation, the samples were dehydrated. After dehydration, lung samples were embedded in optimal cutting temperature compound (Sakura Fintech) and frozen in −80 °C. Samples were cross-sectioned in 10 μm thickness and fixed frozen section in 4% paraformaldehyde for 30 min. After fixation, the slides were stained in Mordant in Bouin’s Solution, 60 °C for 1 h, Weigert’s working hematoxylin for 7 min. Phosphotungstic/phosphomolybdic Acid Solution for 10 min, 1% acetic acid for 1 min sequentially. After staining, the Slides were dehydrated with two changes of 95% ethanol and two changes of 100% Ethanol (2 min per change). Clear with three changes of xylene (5 min per change) and coverslip with permount mounting solution. Light microscopic analysis of lung specimens was performed by blinded observation to evaluate pulmonary fibrosis.

### TUNEL Assay.

Cryopreserved lung tissues were cross-sectioned in 10 μm thickness and fixed with 4% Paraformaldehyde solution (Biosesang, Korea) in PBS (pH 7.4) for 20 min at room temperature. After fixation, the Slides were permeabilized with 0.1% Triton X-100 in 0.1% sodium citrate, freshly prepared, for 2 min on ice. And rinsed slides twice with PBS (pH 7.4), Dry area around samples, add 50 μL TUNEL reaction mixture (11684795910, Roche) on lung tissues, incubated for 60 min at 37 °C in a humidified atmosphere in the dark. After incubation, the Slides were rinsed three times with PBS and the slides were mounted with fluorescence medium (Vector Laboratories) and analyzed using a fluorescence microscope (Leica Microsystem, Germany).

### LAL Assay.

Before running the assay, the Proteins were dissolved at the concentrations of 0, 10, 20, 50, 100, 200, and 500 ng/mL with pyrogen-free water, supplied with the kit. rTOX protein was assayed using a commercially available chromogenic endotoxin kit (A39552, ThermoFisher), according to the manufacturer’s protocol. LPS was detected by LAL, which is catalytically activated enzyme releasing p-nitroaniline (pNA) from the colorless chromogenic substrate, Ac-Ile-Glu-AlaArg-pNA, producing a yellow color. After stopping the reaction, the released pNA is photometrically measured at 405 nm.

### ELISA for Human Plasma CTLA4, PD-1, PD-L1.

The concentrations of CTLA4, PD-1, and PD-L1 in SARS-CoV-2 patients’ plasma were quantified according to the manufacturer’s instructions using a commercially available ELISA kit. Values were measured using an ELISA plate reader (Tecan, Austria GmbH, Austria). Human CTLA4 ELISA kit (ARG82605, Arigo biolaboratories), Human PD-1 ELISA kit (ARG81360, Arigo biolaboratories), Human PD-L1 ELISA kit (ARG83029, Arigo biolaboratories).

### ELISA for TOX Release in Blood Immune Cell.

The blood was obtained from healthy individuals. And Pan T cells, monocytes, Neutrophils were isolated using MACS Pan T cell isolation kit (130-096-535, Miltenyi Biotec), Classical monocyte isolation kit (130-117-337, Miltenyi Biotec), MACSxpress Whole blood neutrophil isolation kit (130-104-434, Miltenyi Biotec), respectively. After isolation, LPS (100 ng/mL) was treated in each cell for 0, 2, 4, 6, 12, and 24 h. After incubation, cell supernatants were harvested, and we performed competitive ELISA using antibodies that recognize human TOX (99036, Cell signaling) The high purity rTOX protein used in our experiments was obtained from Abcam (catalog number ab160644) and is a full-length protein with 526 amino acids (UniProtKB accession number: NP_055544). rTOX (1 μg/100 μL dilution) or plasmas were coated onto Nunc-Immuno™ MicroWell™ 96 well plates and incubated overnight at 4 °C. Prior to use, the plates were washed three times with PBST and blocked with 3% BSA in PBS for 30 min 37 °C. Primary antibody (1:2,000 dilution) and plasma sample (20 μg) were preincubated for 1 h at 37 °C, and then, the preincubated sample was transferred to peptide-coated plate and incubated for 1 h at 37 °C. The plate was washed five times with PBST 5 secondary antibody (1:5,000 dilution) was incubated for 30 min at 37 °C, and then, the plate was washed five times with PBST. The washed plate was treated with TMB ELISA substrate 100 μL/well for 10 min 37 °C and then Stop Solution 100 μL/well was added. The detection was performed at 450 nm by microplate reader (TECAN).

### Assessment of Pulmonary Function Using the FlexiVent System.

C57BL/6 mice were anesthetized by intraperitoneal injection with 120 mg/kg of ketamine and 20 mg/kg of xylazine. Anesthesia was confirmed by a lack of response to noxious stimuli. Mice were subsequently placed on a heated table to maintain physiological body temperature. Orotracheal intubation was performed using an 18 G metal cannula (Scireq, Canada). A suture was carefully applied around the trachea wall to ensure secure placement of the cannula. Mice were then connected to the FX2 device (FlexiVent, Scireq, Canada) for mechanical ventilation. Mice were ventilated at a respiratory rate of 150 breaths per minute, a tidal volume of 0.4 mL/kg, and a positive end-expiratory pressure set at 3 cm H2O. To determine IC, a “Deep Inflation” protocol was executed. This protocol gradually inflated the lung for 3 s to a pressure of 30 cm H2O and maintained this pressure for an additional 3 s to allow for alveolar pressure equilibration. The Forced Oscillation Perturbation test, known as Prime-8, was conducted. This 8 s measurement applied a volume-driven standardized oscillatory frequency test signal, encompassing frequencies both above and below the subject’s ventilation frequency. Different frequencies probed distinct lung regions. Testing of lung mechanical properties, including dynamic compliance, elastance, tissue elasticity, IC, total lung capacity, and quasistatic compliance was carried out by a software-generated script that took four readings per animal.

### Statistical Analysis.

All the in vitro and in vivo data were analyzed via two-tailed unpaired *t*-test using GraphPad prism 7 software, the prepared sample sizes were n ≥ 3, and the statistical significance was set at *P* < 0.05. More detailed information for each experiment is provided in the figure legend. All data normalization processes were carried out according to the manufacturer’s protocol. Data transformation and evaluation of outliers were not used in our study.

## Supplementary Material

Appendix 01 (PDF)

## Data Availability

All study data are included in the article and/or supporting information.
