# Supplementary material for: The TOX–RAGE axis mediates inflammatory activation and lung injury in severe pulmonary infectious diseases
Source: Proc Natl Acad Sci U S A. 2024 Jun 20;121(26):e2319322121. doi: 10.1073/pnas.2319322121 (PMC11214053; doi:10.1073/pnas.2319322121)
Supplement: Supplementary file 1 — Appendix 01 (PDF) [file pnas.2319322121.sapp.pdf]

Supporting Information for

TOX-RAGE axis mediates inflammatory activation and lung injury in severe pulmonary infectious diseases

Hyelim Kim[1,2†], Hee Ho Park[3,4†], Hong Nam Kim[1,5,6,7†], Donghyuk Seo[8], Kyung Soo Hong[9], Jong Geol Jang[9], Eun U Seo[1,5], In-Young Kim[10], So-Young Jeon[10], Boram Son[3], Seong-Woo Cho[2], Wantae Kim[10,*], June Hong Ahn[9,*], and Wonhwa Lee[8,*]

[1]Brain Science Institute, Korea Institute of Science and Technology (KIST), Seoul, 02792 Republic of Korea

[2]Department of Biotechnology, Yonsei University, Seoul, 03722 Republic of Korea

[3]Department of Bioengineering, Hanyang University, Seoul, 04763 Republic of Korea

[4]Research Institute for Convergence of Basic Science, Hanyang University, Seoul 04763, 18 Republic of Korea

[5]Division of Bio-Medical Science and Technology (KIST School), Korea University of Science and Technology (UST), Seoul, 02792 Republic of Korea

[6]School of Mechanical Engineering, Yonsei University, Seoul, 03722 Republic of Korea

[7]Yonsei-KIST Convergence Research Institute, Yonsei University, Seoul, 03722 Republic of Korea

[8]Department of Chemistry, Sungkyunkwan University, Suwon, 16419 Republic of Korea

[9]Division of Pulmonology and Allergy, Department of Internal Medicine, College of Medicine, Yeungnam University and Regional Center for Respiratory Diseases, Yeungnam University Medical Center, Daegu 42415 Republic of Korea

[10]Department of Life Science, University of Seoul, Seoul, 02504, Republic of Korea

[†]H.K., H.H.P., and H.N.K. contributed equally to this work

***Shared Corresponding Authorship:** Wantae Kim, June Hong Ahn, and Wonhwa Lee

Email: wantaekim@uos.ac.kr, fireajh@yu.ac.kr, wonhwalee@skku.edu

This PDF file includes:

Figures. S1 to S12

Table S1

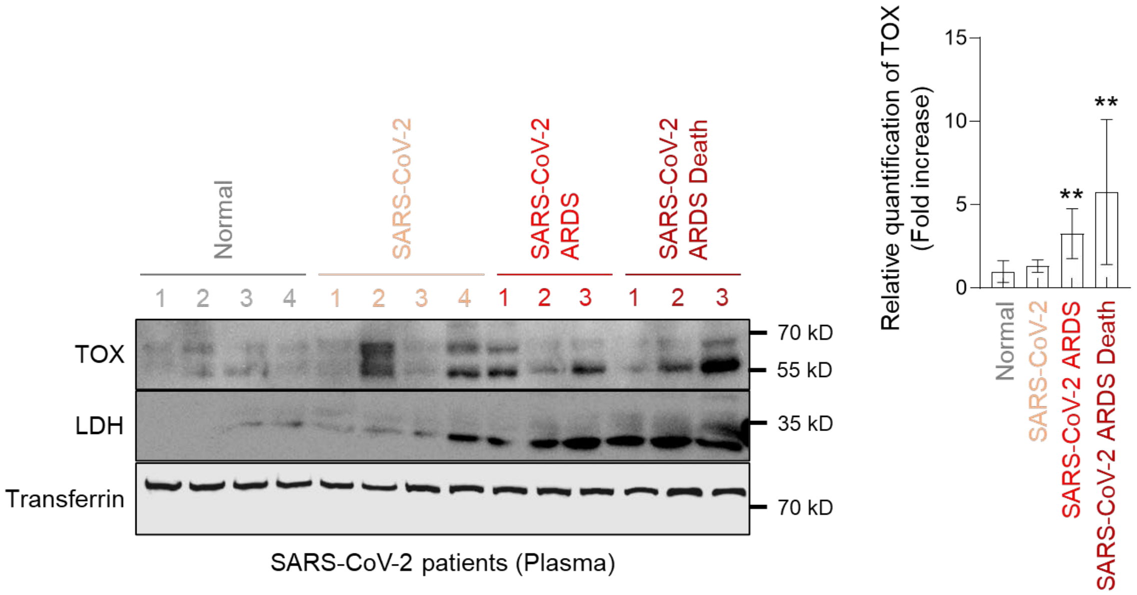

Fig. S1. Western blot analysis of TOX and LDH level in the plasma of SARS-CoV-2 pneumonia patients. The severity of SARS-CoV-2 pneumonia is indicated by the levels of TOX and LDH. Transferrin was used as a serum loading control.

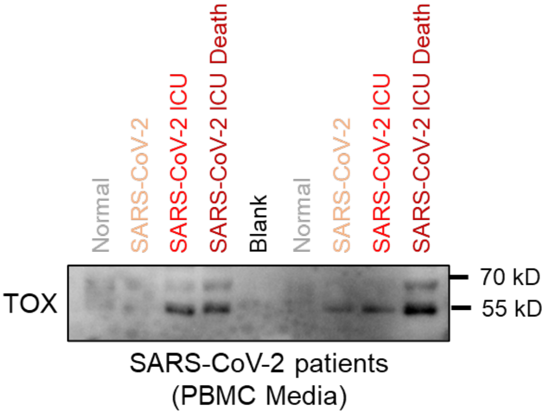

Fig. S2. Western blot analysis of TOX in the PBMC culture media. PBMC was obtained from SARS-CoV-2 patients.

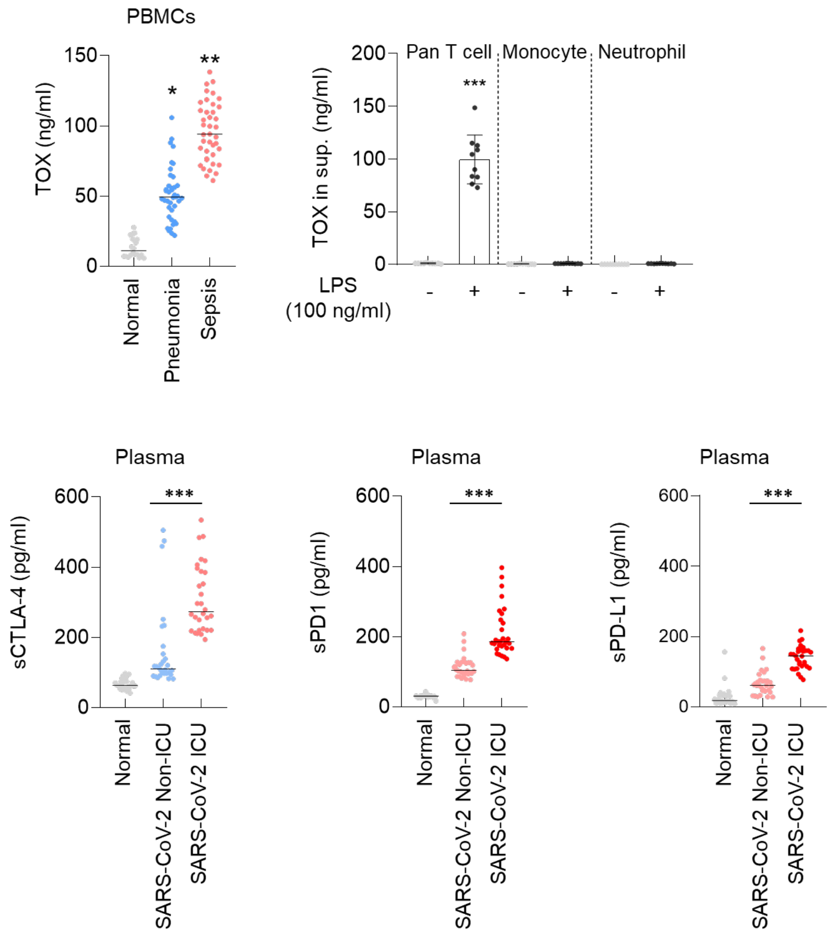

Fig. S3. Level of TOX secreted by pneumonia or sepsis patients PBMCs (*p<0.05, **p<0.01 and, ***p<0.001). Quantification of TOX protein from PBMCs was performed by using ELISA assay (Top left). Quantification of TOX protein from Pan T cells, monocytes, and neutrophils was performed by using ELISA assay (Top right). Quantification of soluble sCTLA-4, sPD-1, sPD-L1 proteins in the sera of COVID-19 patients was performed by using ELISA assay (Bottom). A Student unpaired, two-tailed *t* test was used for comparison of between group data.

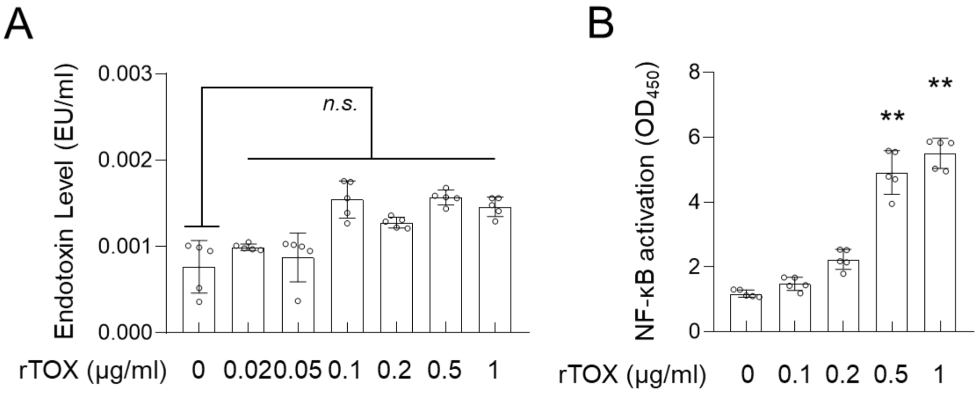

Fig. S4. Response of normal PBMCs to exogenous addition of rTOX. (A) Examination of endotoxin contamination via LAL assay, (B) Activation of NF-κB signaling. (**$p<0.01$). A Student unpaired, two-tailed *t* test was used for comparison of between group data.

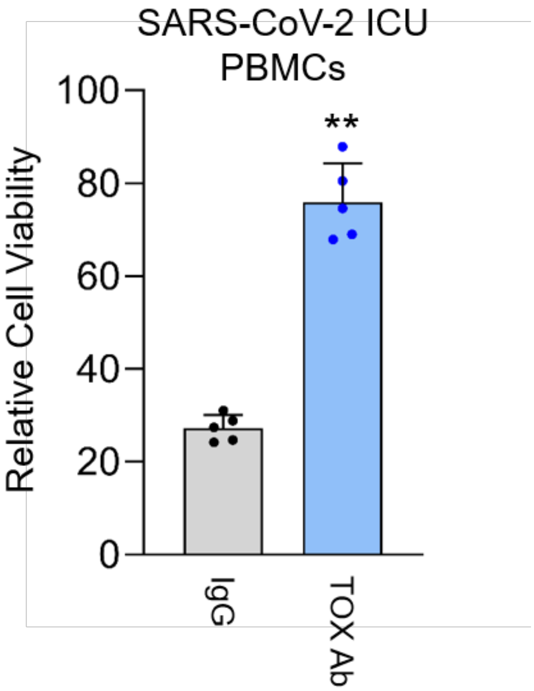

Fig. S5. Relative cell viability of SARS-CoV-2 ICU patients' PBMCs upon the exogenous addition of TOX antibody (**$p<0.01$). A Student unpaired, two-tailed *t* test was used for comparison of between group data.

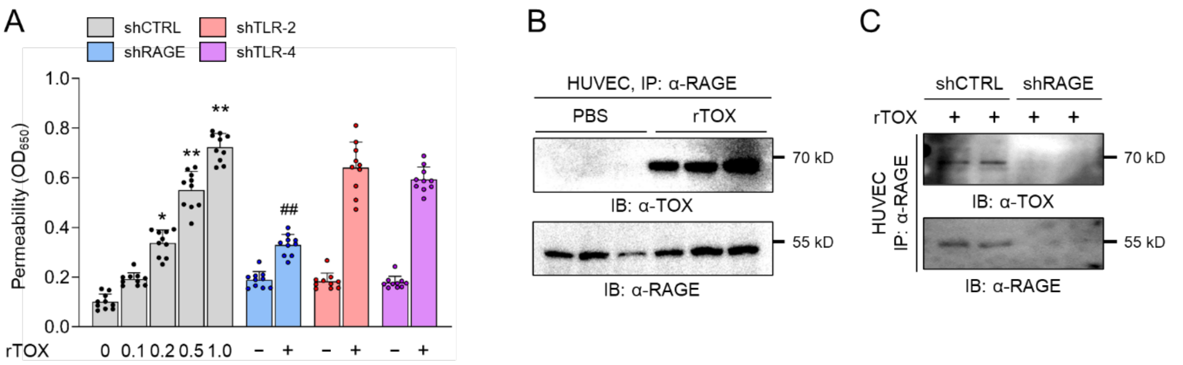

Fig. S6. TOX-RAGE axis induces vascular barrier disruption. (A) Effect of RAGE, TLR-2, and TLR-4 knockdown in the vascular damage. (**p<0.01 and ##p<0.01). A Student unpaired, two-tailed *t* test was used for comparison of between group data. (B, C) RAGE immunoprecipitated with TOX. HUVECs or RAGE knockdown HUVECs were incubated with TOX for 12 hours, and then cell lysates were immunoprecipitated with anti-RAGE.

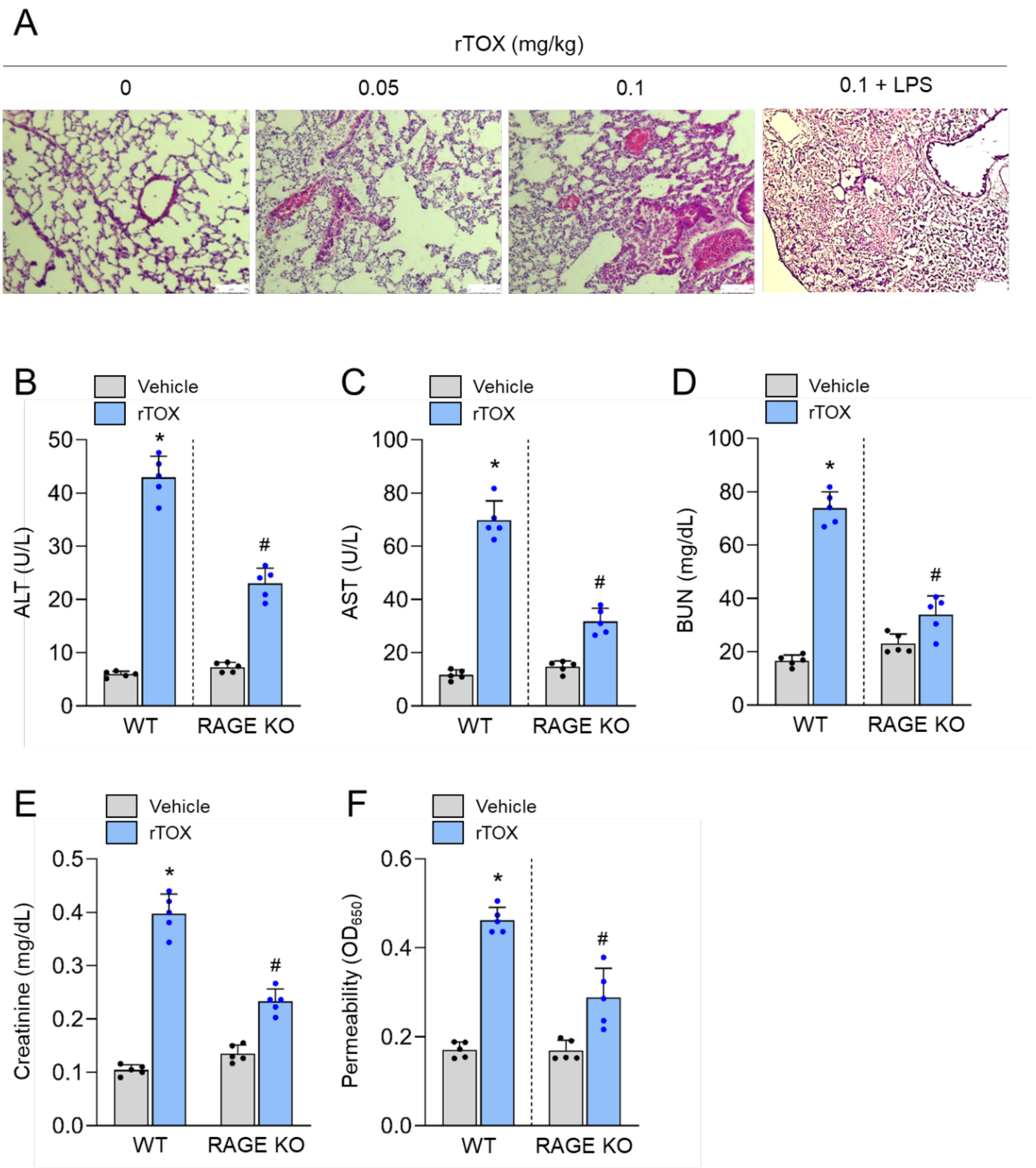

Fig. S7. (A) Effect of injected rTOX concentration on lung tissue damage by using histology and histological score analysis (Scale bar: 75 μm). (B-E). Expression of tissue damage markers in the mouse lung tissue. (B) ALT, (C) AST, (D) BUN, and (E) creatinine. (F) Effect of RAGE knockout in the vascular permeability (#p<0.05, *p<0.01). A Student unpaired, two-tailed *t* test was used for comparison of between group data.

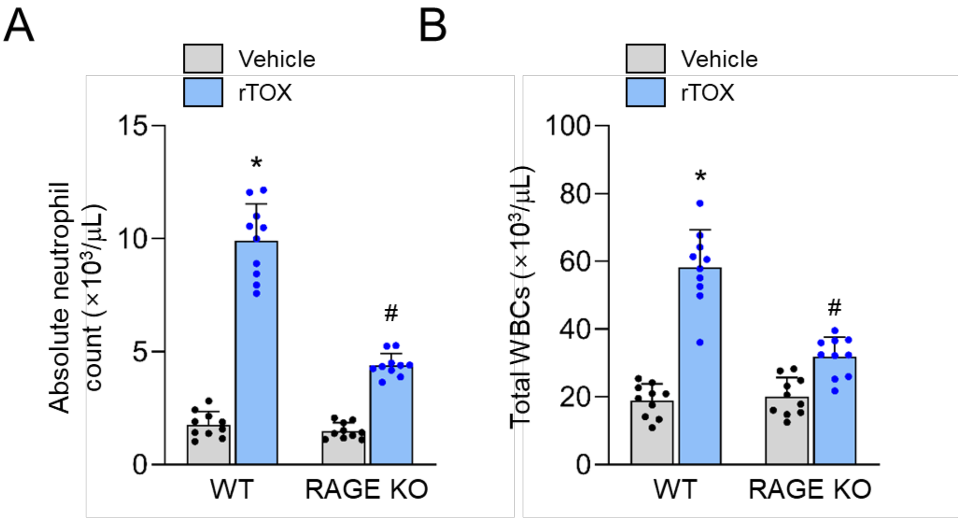

Fig. S8. Quantified mean of neutrophiles and white blood cells in rTOX-injected mouse. Cell counts (cells x 10^3/μL) are presented as (A) absolute neutrophil count, and (B) total white blood cell (WBC) count. (#p<0.05, *p<0.01). A Student unpaired, two-tailed *t* test was used for comparison of between group data.

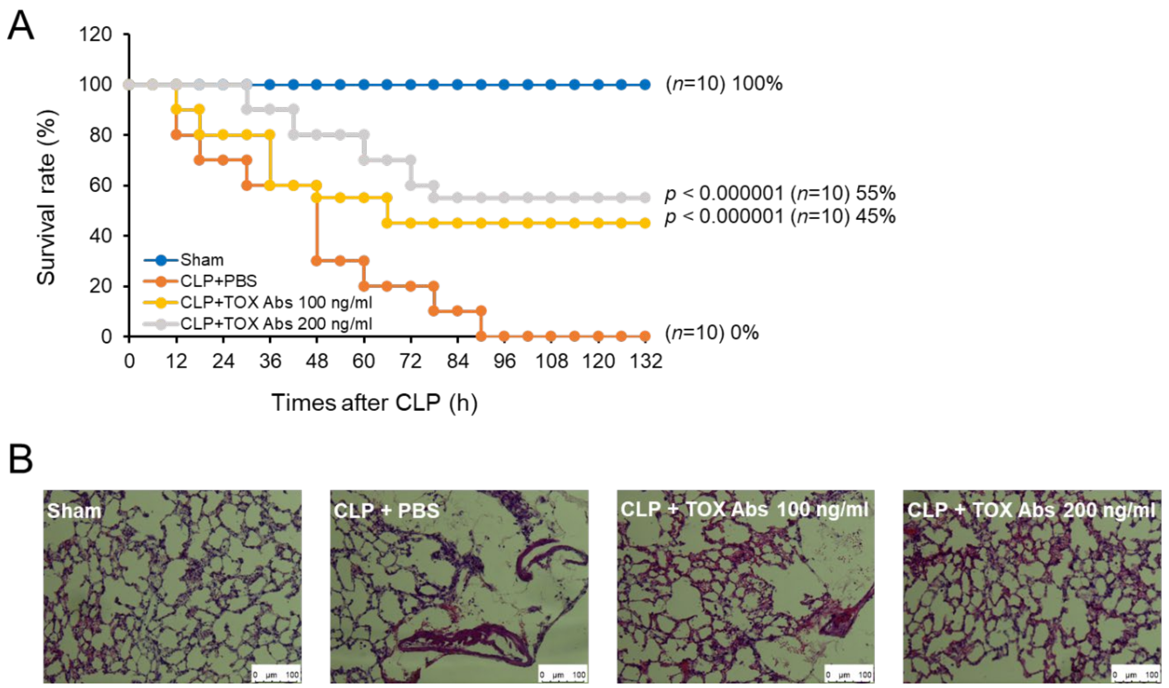

Fig. S9. TOX antibody increases the survival rate and reduces lung tissue damage in septic mice model. (A) Survival rate of CLP-operated mice after the intravenous injection of TOX antibody. (n = 10/each group). (B) Effect of injected TOX antibody concentration on inhibition of lung tissue damage by using histology and histological score analysis. Hematoxylin and Eosin (H&E) staining of CLP-operated mice' lung tissue. Representative images from each group are shown (n = 5). Scale bar, 100 μm. A Student unpaired, two-tailed *t* test was used for comparison of between group data.

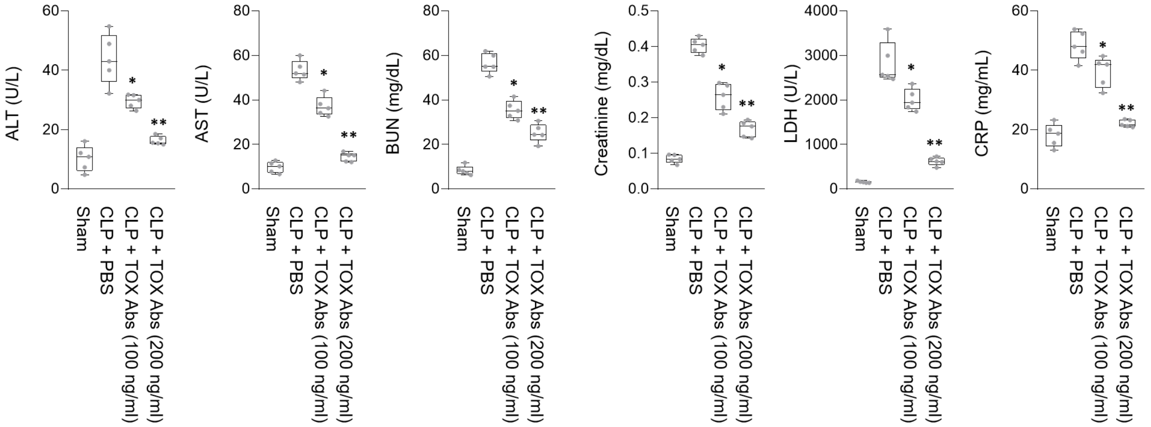

Fig. S10. Concentration of tissue damage markers (alanine aminotransferase, ALT; aspartate aminotransferase, AST; blood urea nitrogen, BUN; creatinine; lactate dehydrogenase, LDH; C-reactive protein, CRP). (n = 5/each group, *p < 0.05, **p < 0.01). A Student unpaired, two-tailed *t* test was used for comparison of between group data.

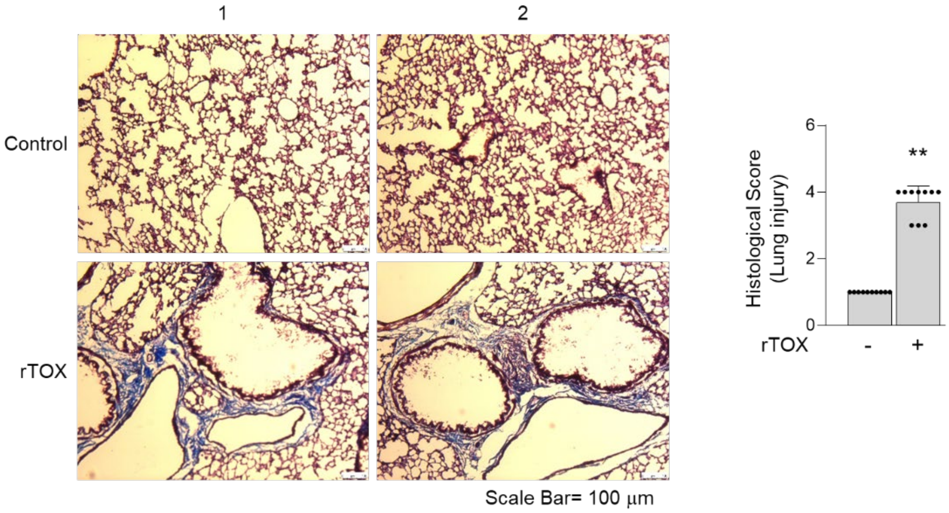

Fig. S11. Histology and histological score of mouse lung tissue 7 days after the intratracheal injection of 0.1 mg/kg rTOX. Lung epithelial cell damage in mice after administration of rTOX. Fibrosis was analyzed via Masson's trichrome staining of lung tissue sections for collagen 1 deposition. (Scale bar: 100 μm).

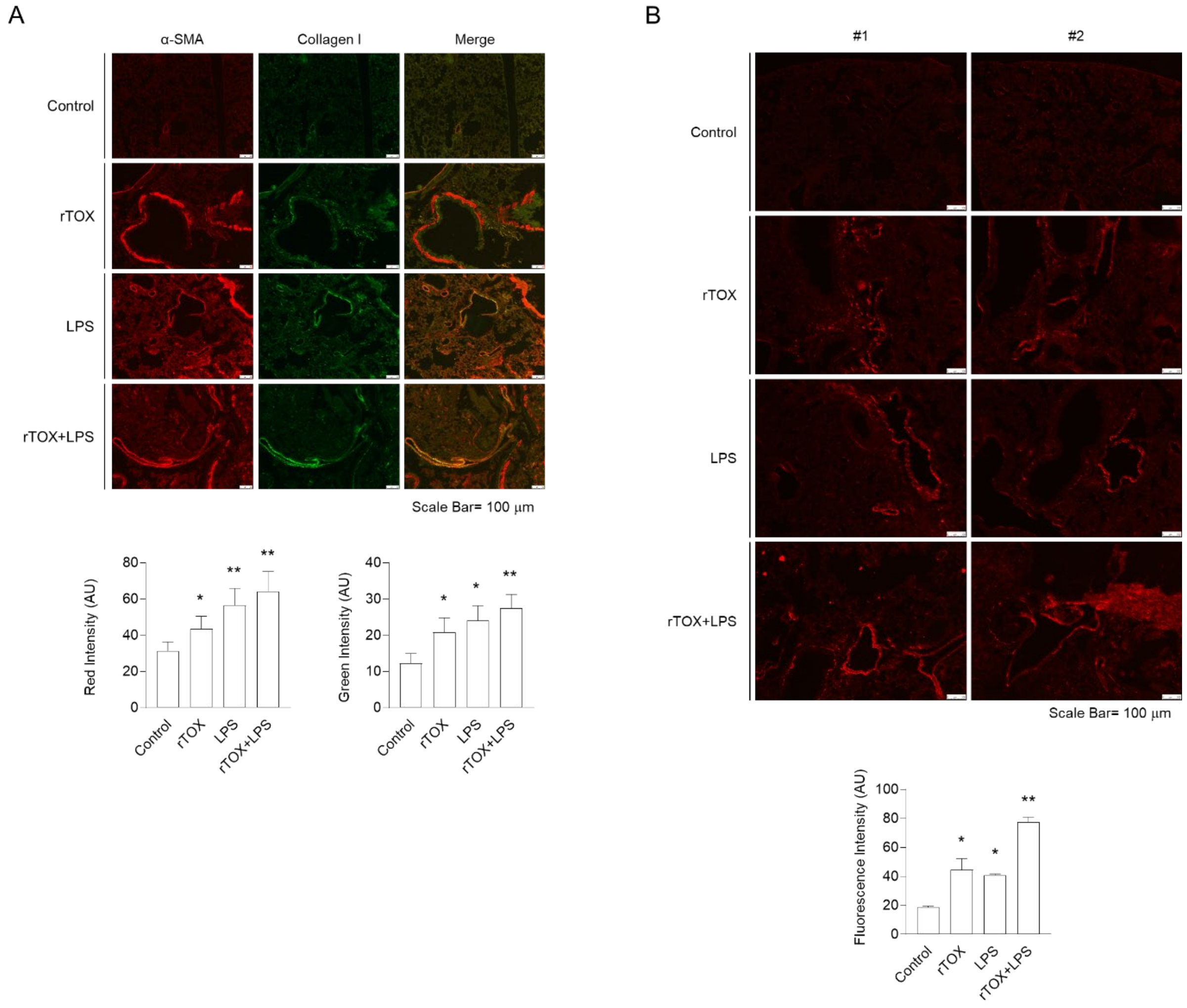

Fig. S12. Histology and histological score of mouse lung tissue 7 days after the intratracheal injection of 0.1 mg/kg rTOX and 5 mg/kg LPS. Lung epithelial cell damage in mice after administration of rTOX. (A) α-SMA and collagen I for lung fibrosis and (B) TUNEL staining for apoptosis of lung tissue sections were analyzed. (Scale bar: 100 μm).

Table S1. Comparison of laboratory findings of patients with SARS-CoV-2 infection

	SARS-CoV-2 Non-ICU (n=85)	SARS-CoV-2 ICU (n=31)	Discharged (n= 31)	P-value
**TOX level in plasma**	90.9 ± 33.2	223.2 ± 33.1	65.9 ± 9.5	<0.001
**Complete blood count**				
White blood cell count, $\times 10^9$/L	6.1 ± 3.2	8.9 ± 3.3	5.6 ± 1.1	0.001
Neutrophil count, $\times 10^9$/L	4.1 ± 3.2	7.7 ± 3.3	3.0 ± 0.9	<0.001
Lymphocyte count, $\times 10^9$/L	1.5 ± 0.7	0.8 ± 0.3	1.9 ± 0.5	<0.001
Hemoglobin, g/dL	13.0 ± 1.6	13.5 ± 1.7	12.6 ± 1.5	0.143
Platelets, $\times 10^9$/L	245.0 ± 107.9	186.7 ± 64.9	258.6 ± 75.0	0.069
**Blood chemistry**				
Albumin, g/dL	3.9 ± 0.5	3.0 ± 0.3	4.0 ± 0.4	<0.001
Alanine aminotransferase, IU/L	30.1 ± 26.3	58.8 ± 93.6	38.8 ± 26.1	0.033
Aspartate aminotransferase, IU/L	37.5 ± 26.6	100.3 ± 97.0	28.4 ± 9.3	<0.001
Total bilirubin, mg/dL	0.8 ± 0.4	1.1 ± 0.6	1.4 ± 4.3	0.398
Blood urea nitrogen, mg/dL	14.6 ± 9.0	20.0 ± 11.4	10.8 ± 3.1	0.001
Creatinine, mg/dL	0.8 ± 0.5	1.0 ± 0.3	0.7 ± 0.2	0.107
Creatinine phosphokinase, IU/L	100.7 ± 159.1	131.9 ± 122.0	71.1 ± 68.3	0.332
Lactate dehydrogenase. IU/L	555.5 ± 184.0	1272.6 ± 542.1	380.4 ± 131.8	<0.001
**Infection biomarkers**				

C-reactive protein, mg/dL	4.2 ± 6.7	17.7 ± 9.5	0.4 ± 1.1	<0.001

Data are presented as mean ± standard error of the mean. (one-way ANOVA).

Table S1. Analysis of the blood of SARS-CoV-2 pneumonia patients. The number of blood cells, tissue damage-related biomarkers were quantified. The number of white blood cells, neutrophils, lymphocytes were compared between the ICU patients, non-ICU and discharged cases. Furthermore, the markers of tissue damage including ALT, AST, BUN, creatinine, LDH, and CRP were also compared between the ICU patients, non-ICU and discharged cases.
